# A cell-specific regulatory region of the human ABO blood group gene regulates the neighborhood gene encoding odorant binding protein 2B

**DOI:** 10.1038/s41598-021-86843-6

**Published:** 2021-04-01

**Authors:** Rie Sano, Yoichiro Takahashi, Haruki Fukuda, Megumi Harada, Akira Hayakawa, Takafumi Okawa, Rieko Kubo, Haruo Takeshita, Junichi Tsukada, Yoshihiko Kominato

**Affiliations:** 1 Department of Legal Medicine, Gunma University Graduate School of Medicine, 3-39-22 Showa-machi, Maebashi, 371-8511 Japan; 2 Department of Legal Medicine, Shimane University School of Medicine, Izumo, Japan; 3 Department of Hematology, University of Occupational and Environmental Health, Kitakyushu, Japan

**Keywords:** Gene expression, Gene regulation, Genetic interaction

## Abstract

The human ABO blood group system is of great importance in blood transfusion and organ transplantation. *ABO* transcription is known to be regulated by a constitutive promoter in a CpG island and regions for regulation of cell-specific expression such as the downstream + 22.6-kb site for epithelial cells and a site in intron 1 for erythroid cells. Here we investigated whether the + 22.6-kb site might play a role in transcriptional regulation of the gene encoding odorant binding protein 2B (OBP2B), which is located on the centromere side 43.4 kb from the + 22.6-kb site. In the gastric cancer cell line KATOIII, quantitative PCR analysis demonstrated significantly reduced amounts of *OBP2B* and *ABO* transcripts in mutant cells with biallelic deletions of the site created using the CRISPR/Cas9 system, relative to those in the wild-type cells, and Western blotting demonstrated a corresponding reduction of OBP2B protein in the mutant cells. Moreover, single-molecule fluorescence in situ hybridization assays indicated that the amounts of both transcripts were correlated in individual cells. These findings suggest that *OBP2B* could be co-regulated by the + 22.6-kb site of *ABO*.

## Introduction

**Figure 1:**
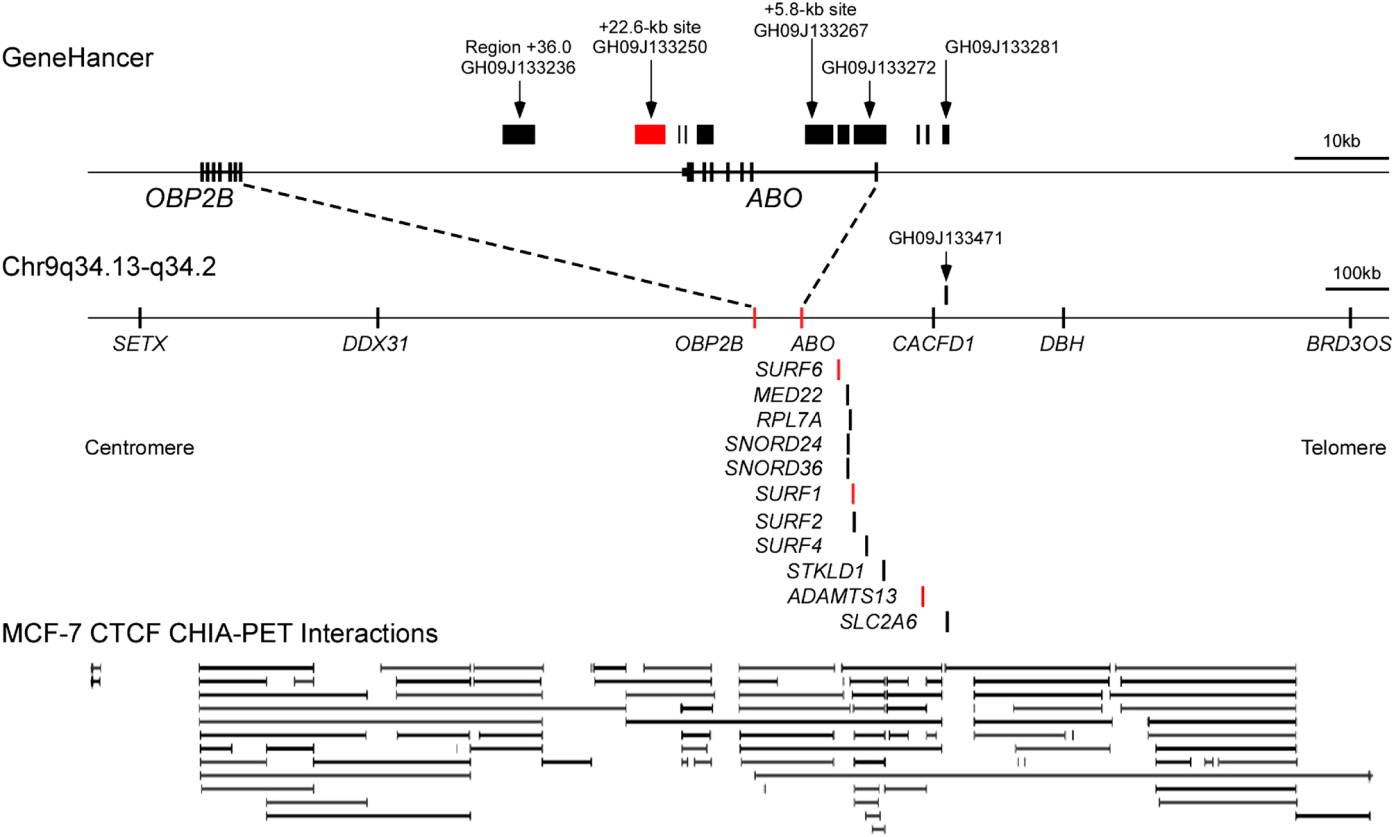
Schematic illustration of the relationships among the genomic regions interacting with the *ABO* transcription start site and the genes around *ABO*. The top diagram represents the genomic regions interacting with the *ABO* transcription start site, which were constructed using publicly available data for GeneHancer Regulatory Elements and Gene Interactions^36^. *ABO* and *OBP2B* are shown below the regions. The *ABO* exons are indicated by lines or a solid box, and the *OBP2B* exons are denoted by lines. The + 5.8-kb site is involved in GH09J133267, the + 22.6-kb site is included in GH09J133250, and the DNase I hypersensitive site region + 36.0 corresponds to GH09J133236^6,7^. The middle diagram shows the chromosomal positions of *ABO* and the other genes shown in Table 1, the transcription start sites of which are indicated by a line. Genes whose transcription start sites interacted with GH09J133250 are colored red. The pseudogene *LCN1P1* is not shown. The bottom represents the locations of CTCF-mediated chromatin interactions on publicly available data for ENCODE Chromatin Interactions tracks involving ChIA-PET data for MCF-7 cells^38^.

The human ABO blood group system is of great importance in blood transfusion and organ transplantation. The carbohydrate structures of ABO blood group antigens are produced by the A- and B-transferases encoded by the *A* and *B* alleles, respectively^1^. The ABO genes are composed of seven exons which span approximately 20 kb of genomic DNA. The difference between the *A* and *B* alleles involves two critical single-base substitutions in exon 7, causing amino acid substitutions that are responsible for the difference in donor nucleotide sugar substrate specificity between the A- and B-transferases^2^. Most *O* alleles are associated with a single base deletion with a frame shift in exon 6, leading to abolition of A-transferase activity. *ABO* transcription is known to be regulated by a constitutive promoter in a CpG island and regions for regulation of cell-specific expression such as the downstream + 22.6-kb site for epithelial cells and the + 5.8-kb site in intron 1 for erythroid cells (Fig. 1)^3–7^. The + 22.6-kb site is located 22.6 kb downstream from the *ABO* transcription start site, and has also been shown to bind the epithelial cell-specific factor Elf5^6^, while the + 5.8-kb site binds transcription factor RUNX1 and the erythroid cell-specific factors GATA-1 and -2^5,8,9^. The functional significance of the promoter and the + 5.8-kb site has been verified by the presence of mutations found in some weak phenotypes such as A_3_, A_m_, B_3_, and B_m_^5,8–14^. It has also been suggested that the transcriptional regulation of *ABO* is responsible for distribution of the A- and B-antigens in a cell-type-specific manner^5,6^, the change in *ABO* expression during cell differentiation^15^, and the decrease of A/B-antigen expression in cancer cells or on red blood cells of patients with leukemia^16–21^.

Individual chromosomes are segregated preferentially into separate territories during interphase^22^. Within these individual chromosome territories, the positioning of genomic regions is correlated with transcriptional activity. One compartment is termed component A (active), and the other is termed compartment B (inactive). These compartments were identified through Hi-C, which is a methodology for measuring the intensities of interaction between any pair of regions in the genome^22^. Another major type of chromatin organization is a self-interacting domain termed the topologically associating domain (TAD) or contact domain^23–25^. In mammalian cells, TAD boundaries are usually demarcated by the chromatin architectural protein CCCTC-binding factor (CTCF) and cohesin^23,24,26–28^. The TAD boundaries preferentially remain stable across cell types, while a small subset of boundaries show cell-type specificity^23,29,30^. In addition, the two interacting DNA sites bound by the CTCF protein and occupied by the cohesin complex form chromosome loop structures: some TADs involve a single loop, while others include multiple loops^31^. These loops frequently contain more than one gene, a feature which could facilitate the co-regulation and co-expression of genes located within the same loop^32–34^. Recently, Giammartino et al. reported that disruption of the KLF4 binding site within the *Tbx3* enhancer weakened enhancer-promoter contacts and diminished the expression of *Tbx3*, *Gm16063* and *Aw549542* in pluripotent stem cells^35^.

In the present study using the gastric cancer cell line KATOIII, as the gene encoding odorant binding protein 2B (OBP2B) is located on the centromere side 43.4 kb from the + 22.6-kb site (Fig. 1), we demonstrated that the *OBP2B* transcripts were reduced in mutant cells with biallelic deletions of the + 22.6-kb site which were created by the CRISPR/Cas9 system, relative to the amounts of transcript in the wild-type cells. Moreover, single-molecule fluorescence in situ hybridization assays indicated that the amounts of both *ABO* and *OBP2B* transcripts were correlated in individual cells. These observations appear to provide new insight into the network of gene regulation between *ABO* and *OBP2B* in the neighbourhood of *ABO*.

## Results

### Involvement of the + 22.6-kb site of *ABO* in transcriptional regulation of *OBP2B*

Publicly available data for GeneHancer Regulatory Elements and Gene Interactions on the UCSC Genome Browser indicated that region GH09J133250 including the + 22.6-kb site interacted with transcription start sites of genes such as *OBP2B*, *LCN1P1*, *SURF6*, *SURF1*, and *ADAMTS13* around *ABO* (Fig. 1, Table 1)^36,37^. In addition, publicly available data derived from chromatin interaction analysis by paired-end tag sequencing (ChIA-PET) on the UCSC Genome Browser indicated that *OBP2B, ABO*, and the + 22.6-kb site were included within most CTCF-anchored loops formed around *OBP2B* or *ABO* in the breast cancer cell line MCF-7, which is representative of epithelial cells, whereas *SURF6*, *SURF1*, *ADAMTS13*, *ABO*, and the + 22.6-kb site were involved within a few CTCF-anchored loops (Fig. 1)^38^. This suggested that the + 22.6-kb site might preferentially regulate *OBP2B* rather than *SURF6*, *SURF1*, and *ADAMTS13*.

**Table 1:** Genomic regions interacting with the transcription start sites of *ABO*.

Genomic regions of GeneHancer ID^a^	Position^b^		Genes whose transcription start site interacted with a region in the left column^c^	
GH09J133471	− 186,658	− 185,332		*SURF1, SURF4, CACFD1, SLC2A6*
GH09J133281	− 7689	− 6962	*DDX31*	*SURF6, SURF4, STKLD1, ADAMTS13*
GH09J133280	− 5545	− 5396		*SURF6, SURF1, SURF4, STKLD1*
GH09J133279	− 4536	− 4471	*LCN1P1*	*SURF6, SURF4, STKLD1*
GH09J133272	− 1250	+ 2265	*DDX31*	*SURF6, CACFD1*
GH09J133271	+ 2702	+ 3927		*SURF6*
GH09J133267	+ 4364	+ 7201		*SURF6, MED22, RPL7A, SNORD24, SNORD36, SURF1, SURF2, DBH*
GH09J133256	+ 16,767	+ 18,458		
GH09J133254	+ 20,500	+ 20,841	*OBP2B*	
GH09J133253	+ 21,041	+ 21,251	*OBP2B*	
GH09J133250	+ 21,708	+ 24,882	*OBP2B, LCN1P1*	*SURF6, SURF1, ADAMTS13*
GH09J133236	+ 35,312	+ 38,609	*SETX, EEF1A1P5* *OBP2B, LCN1P1*	*MED22, RPL7A, BRD3OS*

^a^Genomic regions interacting with the *ABO* transcription start site are listed on the basis of publicly available data of GeneHancer Regulatory Elements and Gene Interactions^36^.

^b^Positions of interacting regions are denoted relative to the ATG translational start site of *ABO,* chr 9:136,150,605 in human GRCh37/hg19.

^c^Genes whose transcription start sites are interacted with a region in the left column are shown in the right-hand columns, genes present upstream of *ABO* on the telomere side are shown on the right, the genes downstream of *ABO* on the centromere side are shown on the left. *OBP2B* and *LCN1P1* belong to a Lipocalin family, whereas *LCN1P1* is a pseudogene. Positions of *LCN1P1*, *SLC2A6* and *EEF1A1P5* are not shown in Fig. 1.

**Figure 2:**
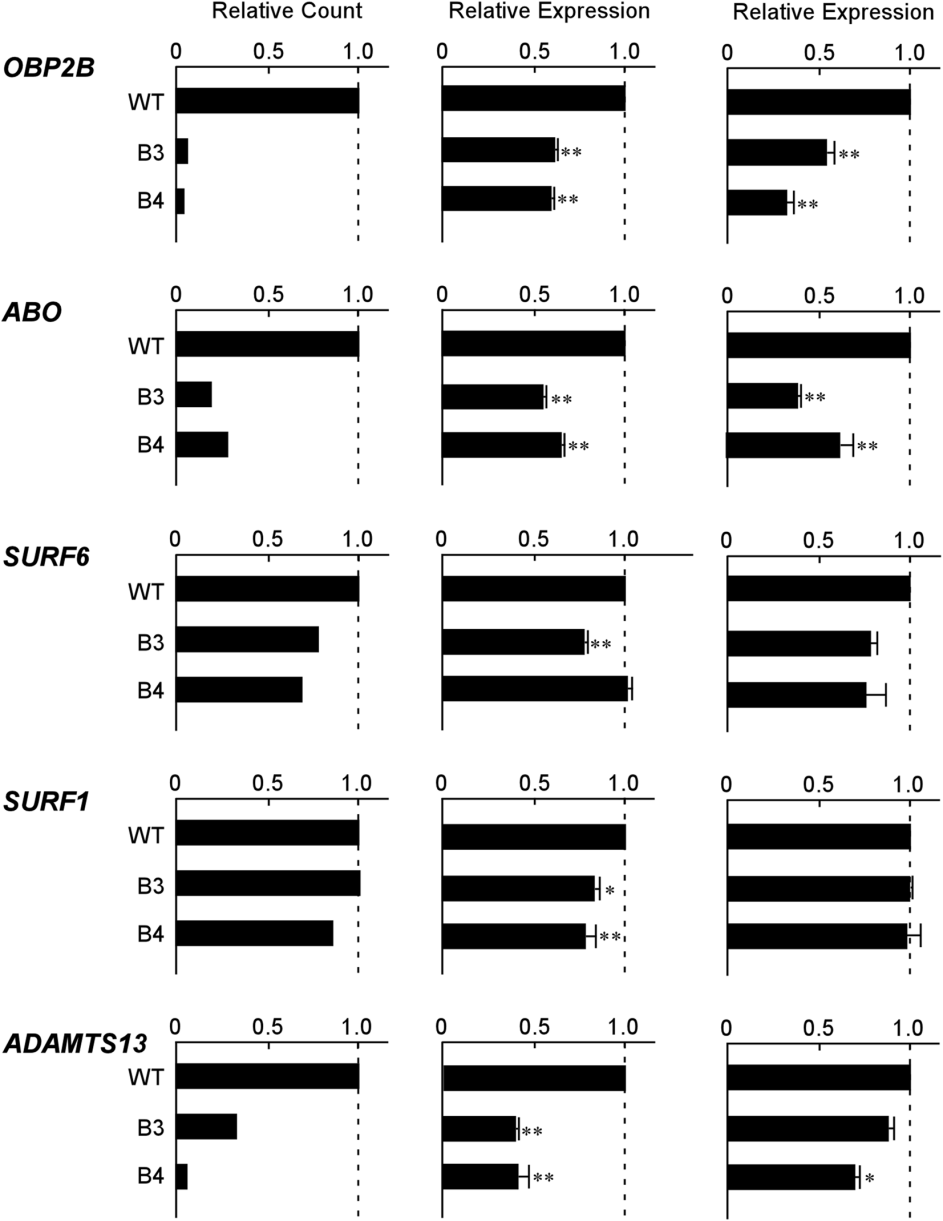
Decrease of *OBP2B* and *ABO* expression in KATOIII cells harboring biallelic deletions of the + 22.6-kb site. Each panel shows the relative amounts of various transcripts including *OBP2B*, *ABO*, *SURF6*, *SURF1*, or *ADAMTS13* in wild-type KATOIII cells and their derived mutant clones B3 and B4 harboring biallelic deletions of the + 22.6-kb site. The left column of panels represents the relative expression of each gene obtained from TMM-normalized counts of RNA-seq in the wild-type and mutant cells. When the count of each transcript in the wild-type cells was assigned an arbitrary value of 1.0, the relative count of each transcript was calculated in the mutant clones. Similarly, the middle or right column of panels represents the relative expression of each gene obtained using real-time PCR or ddPCR, respectively, in the wild-type and mutant cells. The ratio of each target transcript was calculated by dividing it by the copy number of *β-actin* or *18S rRNA* in real-time PCR or ddPCR, respectively. When the ratio of each transcript in the wild-type cells was assigned an arbitrary value of 1.0, the relative expression of each transcript was calculated in the mutant cells. All data represent means from three independent experiments, and the standard deviations are also shown. The significance of differences was determined by Student’s *t* test at a significance level of *p* value < 0.01 (**) or 0.05 (*).

To examine whether the + 22.6-kb site was involved in transcriptional regulation of *OBP2B*, we performed RNA-seq and quantitative PCR using wild-type KATOIII cells and derived clones B3 and B4 harboring biallelic deletions of the + 22.6-kb site created using the CRISPR/Cas9 system^6^. RNA-seq indicated that the biallelic deletions resulted in loss of 94‒96% of the *OBP2B* transcript in B3 and B4 cells relative to the wild-type cells, and in loss of 72‒81% of the *ABO* transcript (Fig. 2). To verify the decrease in the *OBP2B* transcript, real-time PCR was performed, and this demonstrated that the deletions resulted in loss of 39‒41% of the *OBP2B* transcripts and 35‒45% of the *ABO* transcripts (Fig. 2). Droplet digital PCR (ddPCR), which is appropriate for precise determination of the ratio of transcripts between the wild-type cells and mutant cells, demonstrated 46‒68% reduction of the *OBP2B* transcripts and 38‒62% reduction of the *ABO* transcripts in the mutant cells (Fig. 2). Thus, it appeared that the biallelic deletion of the + 22.6-kb site reduced transcription from *OBP2B* and *ABO* in the epithelial cells.

Since the GH09J133250 region is also known to interact with the transcription start sites of genes including *LCN1P1*, *SURF6*, *SURF1*, and *ADAMTS13* other than *OBP2B* and *ABO* (Table 1), quantitative analysis was performed using real-time PCR and ddPCR (Fig. 2). For *SURF6*, a significant decrease in the transcripts was demonstrable only in B3 cells by real-time PCR, whereas ddPCR demonstrated no significant reduction of the transcripts in either B3 or B4 cells. Regarding *SURF1*, a significant reduction of the transcripts was demonstrated by real-time PCR in both mutant cell lines, whereas ddPCR did not indicate a significant decrease in either B3 or B4 cells. Regarding *ADAMTS13*, real-time PCR demonstrated a significant decrease of the transcripts in both mutant cell lines, whereas ddPCR demonstrated a significant decrease in B4 cells but not in B3 cells. Because *LCN1P1* is a pseudogene, quantitative analysis was not carried out. Thus, it was uncertain whether biallelic deletions of the site led to a decrease of *SURF6*, *SURF1*, and *ADAMTS13* transcripts in epithelial cells. In comparison with *ABO* and *OBP2B*, *ADAMTS13* had a lower copy number, while *SURF1* and *SURF6* had higher copy numbers in KATOIII cells (data not shown). Because the amounts of these transcripts were near the lower or upper limit of the quantification range of the corresponding real-time PCR, and ddPCR is an absolute quantification test, this might have contributed to the discrepancies in the results between real-time PCR and ddPCR.

Human *OBP2B* and *OBP2A* are 97.5% identical to each other^39^. To examine whether the qPCRs were specific to *OBP2B*, the real-time PCR products were cloned into a cloning vector, followed by sequencing. Nucleotide determination of 12 clones demonstrated that every PCR product was derived from *OBP2B* on the basis of nucleotide substitutions from c.331 to c.503 in *OBP2B* with a reference sequence of NM_014581.3 where 11 nucleotide substitutions were present between *OBP2B* and *OBP2A*. In addition, RT-PCR was carried out using primers complementary to the consensus sequences for *OBP2B* and *OBP2A* with cDNA prepared from KATOIII cells, followed by cloning and sequencing. Nucleotide determination of 20 clones demonstrated that all products were derived from *OBP2B* on the basis of the nucleotide sequences from c.269 to c.503 in *OBP2B* where 16 nucleotide substitutions were present between *OBP2B* and *OBP2A*. Direct sequencing of the RT-PCR products did not detect nucleotide variants specific to *OBP2A* (data not shown). These results indicated that *OBP2A* was scarcely expressed in KATOIII cells.

### Decrease of OBP2B protein expression in both the cell lysate and supernatant of mutant cells lacking the epithelial cell-specific regulatory region of *ABO*

**Figure 3:**
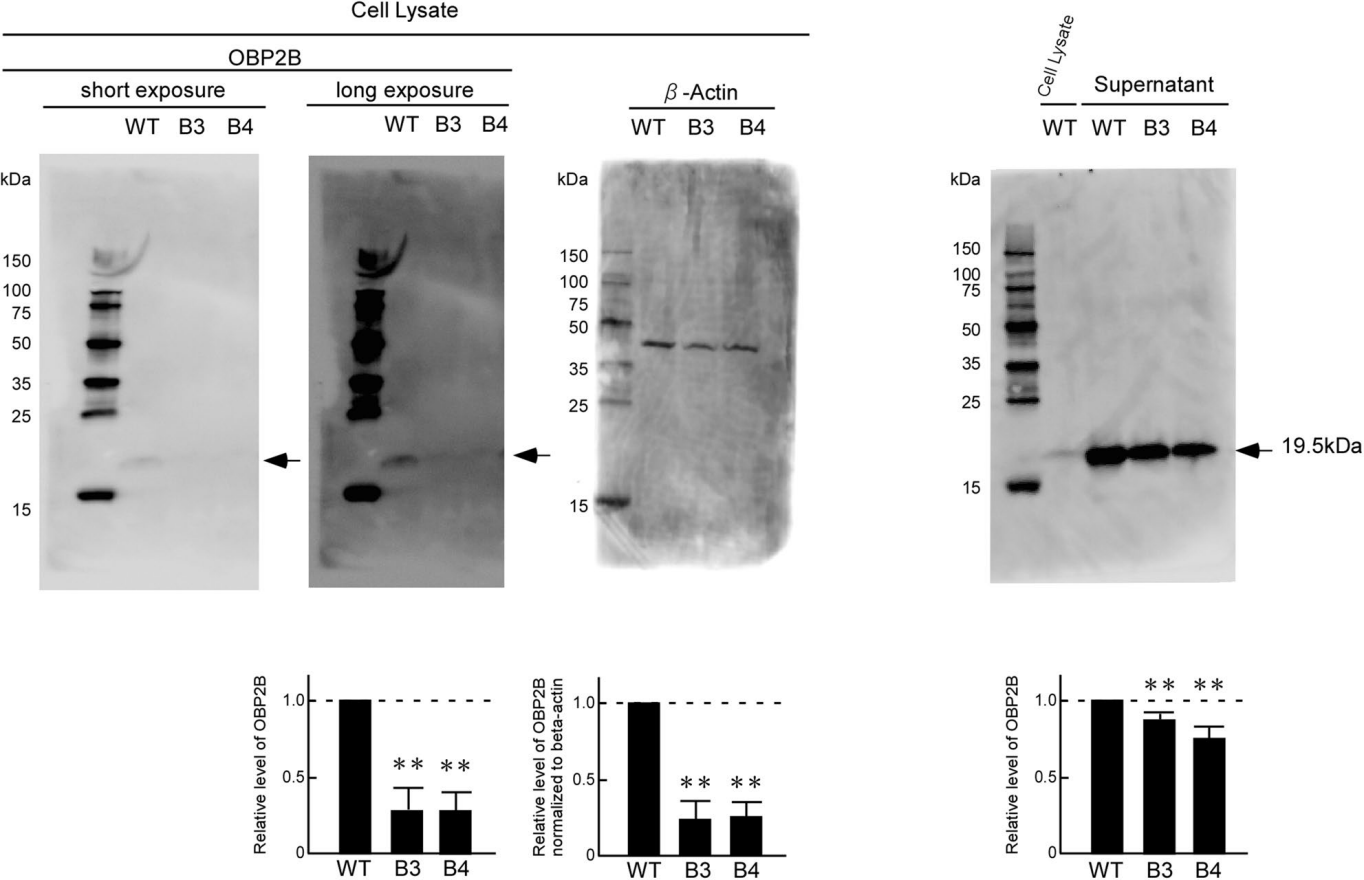
Quantitative decrease of OBP2B protein in the mutant clones B3 and B4 relative to that in the wild-type KATOIII cells. The amount of OBP2B or β-actin protein was evaluated by Western blotting using the cell lysate and supernatant prepared from the wild-type KATOIII cells and its derived mutant clones B3 and B4, followed by densitometry measurements. The representative blots are shown in the upper panels. The two panels on the left side show the blots of OBP2B obtained using a cell lysate after short or long exposure. The molecular weight of each protein was estimated using ECL DualVue Western blotting markers (cytiva). The amount of protein applied to each lane was 30 μg for the cell lysate or 20 μg for the supernatant. The left and right lower panels indicate the relative amounts of OBP2B protein in the cell lysate and supernatant, respectively, obtained from the mutant cells when that of the wild-type cells was assigned an arbitrary value of 1.0. The middle lower panel indicates the level of OBP2B normalized to the amount of β-actin in cell lysates in the mutant cells, relative to that for wild-type cells which was assigned an arbitrary value of 1.0. The relative level of OBP2B represents the mean from more than three independent experiments. The significance of the decrease was determined by Student’s *t* test at a significance level of *p* < 0.01 (**).

To verify that OBP2B protein expression was reduced in the mutant clones B3 and B4 relative to that in wild-type KATOIII cells, Western blotting was performed using the cell lysate and supernatant. This revealed a decrease of OBP2B protein in both the cell extract and supernatant of the mutant cells relative to the wild-type cells (Fig. 3). Therefore, it was likely that the + 22.6-kb site played an important role in transcriptional regulation of *OBP2B* expression in cells of epithelial lineage. However, biallelic deletion of the + 22.6-kb site did not achieve complete loss of the *OBP2B* expression, suggesting additional regulatory regions to be involved in regulation of the expression.

### Examination of *OBP2B* transcriptional regulation

**Figure 4:**
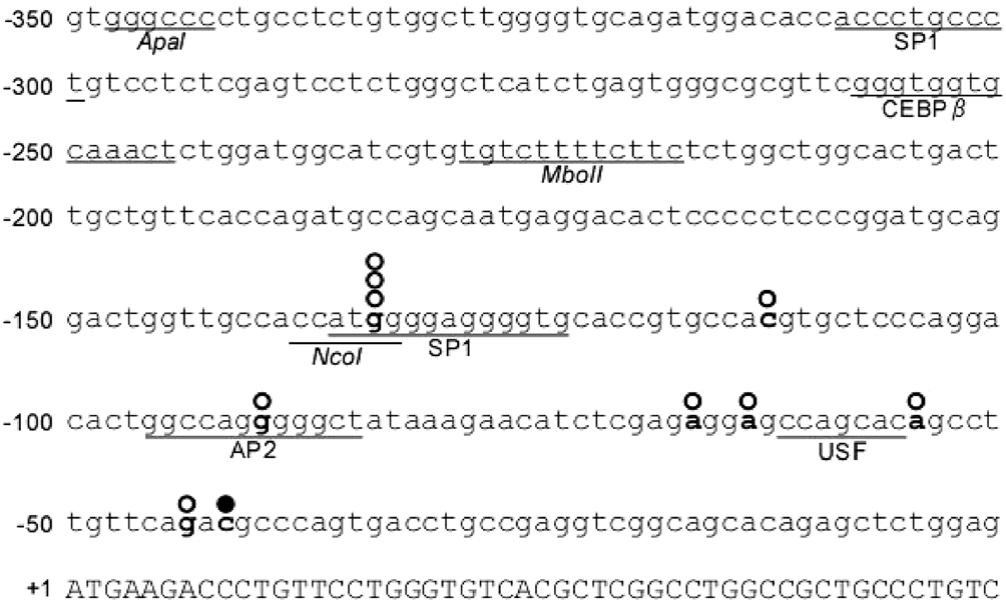
The nucleotide sequence of the 5′-flanking region in the human OBP2B gene. The sequence is given in full, from position − 350 to + 50, relative to the translation start site in exon 1 of *OBP2B*. The uppercase letters denote the coding sequence of exon 1, and the lowercase letters indicate non-coding genomic sequence. Several restriction enzyme recognition sites are underlined. Several putative transcription factor binding sites are underlined. Open circles indicate the locations of the 5′-ends of the *OBP2B* transcripts, determined by 5′-RACE using cDNA obtained from KATOIII cells, while the filled circle denotes the transcription initiation site obtained from the publicly available data for GeneHancer Regulatory Elements and Gene Interactions^36^.

The transcription initiation site as reported on the UCSC Genome Browser was located 42 nucleotides upstream from the translation start site of *OBP2B*^36^. To identify the promoter region of *OBP2B*, 5′-RACE was performed using cDNA synthesized from RNA of KATOIII cells. Agarose gel electrophoresis of the 5′-RACE products demonstrated a major band, and the DNA fragments were purified and cloned into a sequencing vector. The DNA sequences for 9 transformant clones were determined. The 5′-ends of the 5′-RACE products were located from 134‒44 nucleotides upstream from the translation start site of *OBP2B*, although the site at position − 134 was used most frequently as the transcription start site (Fig. 4).

**Figure 5:**
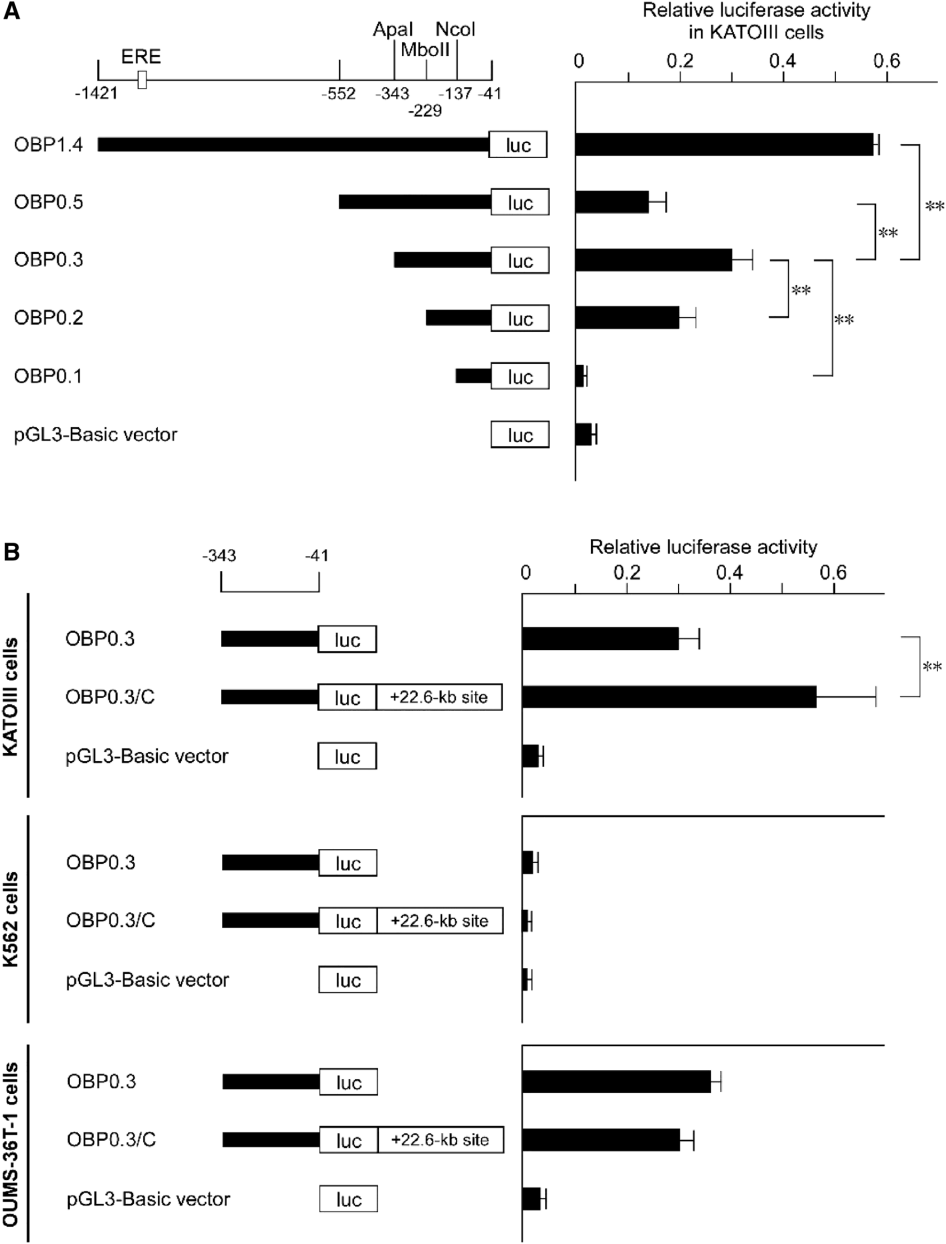
Summary of the relative luciferase activities of the reporter constructs containing different lengths of the 5′ upstream sequence of *OBP2B*. The *OBP2B* sequences (horizontal bars) were inserted upstream of the *luciferase* coding sequence of the pGL3-basic vector. Constructs were aligned below the restriction map of the region and are shown in the left panel. ERE represents location of estrogen response element. Construct names are shown to the left of the bar, and the locations of the inserted fragments are shown. The + 22.6-kb site was inserted downstream of *luciferase* in construct OBP0.3/C. Each construct as depicted on the left was transiently transfected into KATOIII cells in (**A**), and each construct was transiently transfected into KATOIII cells, K562 cells, or OUMS-36T-1 cells in (**B**). The obtained luciferase activity was normalized, and is shown in the right panel. The mean values and standard deviations were calculated from more than three independent experiments. The significance of differences was determined by Student’s *t* test at a significance level of *p* < 0.01 (**). The activity of the pGL3-promoter vector containing the SV40 promoter was given an arbitrary value of 1.0.

In order to examine the transcriptional activity of the 5′ upstream sequences of *OBP2B*, we employed reporter and transfection systems. We first obtained reporter plasmid OBP1.4 by introducing the 1.4-kb genomic fragment 5′-flanking the coding sequence of *OBP2B* into the promoterless pGL3-basic vector upstream from the *luciferase* coding sequence (Fig. 5A). This plasmid was transiently transfected into KATO III cells. The promoter activity of the OBP1.4 construct was at least 15-fold higher than that of the pGL3-basic vector, and twofold lower than that of the pGL3-promoter vector containing the SV40 promoter. Deletion of the *OBP2B* upstream region from position − 1421 to − 552 resulted in a large decrease of luciferase activity, demonstrating that the important sequences for *OBP2B* transcription were contained within the deleted region. Further deletion of the sequence from − 552 to − 343 resulted in an increase of luciferase activity, suggesting that negative element(s) for the *OPBP2B* transcription were present within this deleted region. Furthermore, deletion of the sequence from − 343 to either − 229 or − 137 resulted in a large decrease of luciferase activity, confirming the importance of promoter sequences immediately upstream of the transcription start site (cap site) for *OBP2B* expression. Thus it appeared that the region from − 343 to − 41 was required for the promoter activity of *OBP2B*. Further study will investigate the upstream regions in detail to elucidate the *OBP2B* regulation involving the + 22.6-kb site.

Furthermore, the *OBP2B* promoter was proved to be enhanced by the + 22.6-kb site in KATOIII cells; introduction of the + 22.6-kb site downstream from *luciferase* resulted in an approximately twofold increase of luciferase activity relative to OBP0.3 in KATOIII cells (OBP0.3/C, Fig. 5B). In terms of cell specificity, the promoter was not active in K562 cells. In OUMS-36T-1 cells, the promoter was active, although its activity was not enhanced by the + 22.6-kb site (OBP0.3/C, Fig. 5B). These findings indicated that the *OBP2B* promoter activity was enhanced by the + 22.6-kb site in an epithelial cell-specific manner.

### Involvement of the + 22.6-kb site in co-expression of *ABO* and *OBP2B*

**Figure 6:**
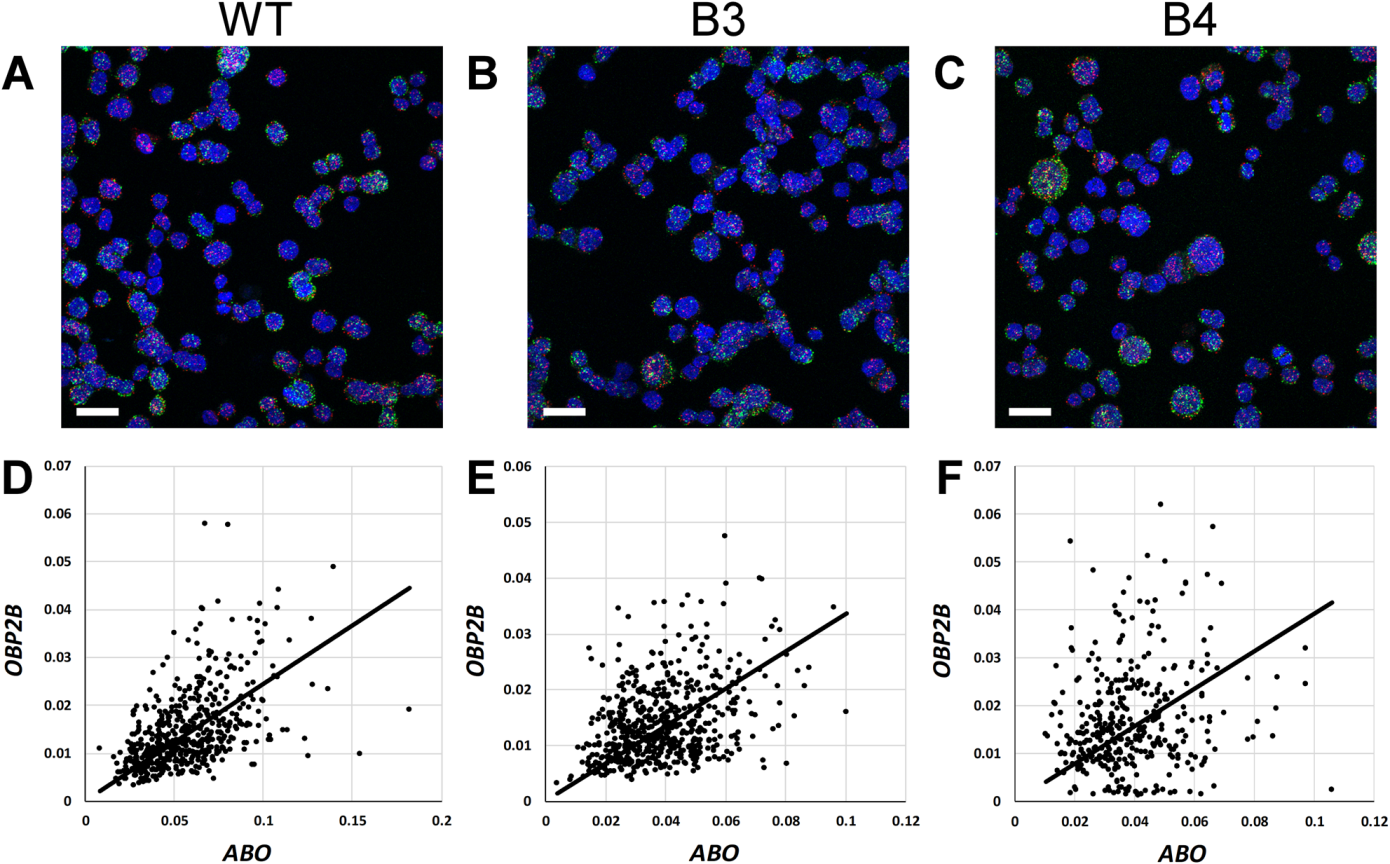
RNA fluorescence in situ hybridization for the *ABO* and *OBP2B* transcripts. (**A**–**C**) Representative images of the wild-type KATOIII cells (**A**), and their mutant clones B3 (**B**) or B4 (**C**) fluorescently labeled for transcripts *ABO* (Opal570; shown in red) and *OBP2B* (Opal520; green), as well as nuclei (DAPI; blue). Scale bar; 20 µm. (**D**–**F**). Mean relative intensity of the *ABO* and *OBP2B* signals in each nucleus for the wild-type cells (**D**), B3 cells (**E**) or B4 cells (**F**). Each dot represents each ROI (nucleus), and the x-axis indicates the mean relative intensity of the Opal570 signal (*ABO*) in each ROI while the y-axis shows that of Opal520 (*OBP2B*). The coefficients of determination (R^2^) were 0.812 for the wild-type cells, 0.802 for B3 cells and 0.666 for B4 cells.

To examine whether the *ABO* transcripts were correlated with the *OBP2B* transcripts, we performed single-molecule fluorescence in situ hybridization assays visualizing both transcripts in individual wild-type KATOIII cells (Fig. 6A). Analysis of more than 500 nuclei of the wild-type cells revealed that the expression of *ABO* was correlated with that of *OBP2B* in single cells with a coefficient of determination (R^2^) of 0.812 (Fig. 6D). Subsequently, to examine whether the + 22.6-kb site might contribute to co-expression of those genes, the hybridization assays were carried out in individual cells of the mutant clones B3 and B4 (Fig. 6B,C). Similar co-expression of *ABO* and *OBP2B* was observed in the mutant cells. Because R^2^ of B3 or B4 was 0.802 or 0.666, respectively (Fig. 6E,F), either was not as high as that of the wild-type cells. However, it remains to be investigated whether biallelic deletion of the + 22.6-kb site would increase the cell-to-cell variation of co-expression.

## Discussion

In the present study, we demonstrated that *OBP2B* expression was reduced in gastric cancer cells with biallelic deletion of the epithelial cell-specific positive regulatory element of *ABO*, or the + 22.6-kb site, and that the *OBP2B* promoter activity was enhanced by the + 22.6-kb site as revealed by transient transfection of luciferase reporter plasmids into the epithelial cells. Therefore, these observations suggested that transcription of *OBP2B* and *ABO* was dependent upon the + 22.6-kb site, which functioned in an epithelial cell-specific manner. Moreover, single-molecule fluorescence in situ hybridization assays indicated a tendency for the two gene-neighborhoods *OBP2B* and *ABO* to have consistent activities, suggesting that genes in these neighborhoods could be co-regulated by the + 22.6-kb site. Long-range chromatin loops that are mediated by CTCF can facilitate enhancer–promoter interactions^31,40^, and could facilitate the co-regulation and co-expression of gene pairs^32–34^. Therefore, it seemed plausible that *OBP2B* and *ABO* were co-regulated by the epithelial cell-specific regulatory region of *ABO*.

Human *OBP2A* and *OBP2B* have been identified on 9q34^39^. Although they are homologous, they are differentially expressed in secretory structures. *OBP2A* is strongly expressed in lachrymal glands, nasal structures, salivary and lung, whereas *OBP2B* is expressed more strongly in such organs as the mammary glands and prostate. They belong to the lipocalin (LCN) family^39^, which includes a diverse group of low-molecular-weight proteins (18‒40-kDa)^41^. The LCNs are conserved through evolution and share an eight-stranded antiparallel β-sheet structure forming a barrel, which is the internal ligand-binding site that interacts with and transports small hydrophobic molecules including odorants, retinoids, steroid hormones, and lipids. Therefore, LCNs play important roles in physiological processes by binding to and transporting these small hydrophobic molecules. Odorant-binding proteins are thought to be secreted, and act by transporting hydrophobic molecules within mucus. The solubilization of odorant is the first step in the process of olfaction in the hydrophilic nasal mucus, since olfaction involves the binding of small, hydrophobic, volatile molecules to receptors of the nasal neuroepithelia, generating a cascade of neurological events that transmit information to the olfactory bulbs projecting into the brain. It is expected that OBP2B would be involved in lactation, since it is produced in the tubulo-acinar secretory cells of the mammary glands where ABH antigens are synthesized^39^. Although the species of odorant or lipid that binds to human OBP2B has remained elusive, it has been shown that rat OBP2 binds some odorous compounds such as chromopore 1-anilinonaphthalene 8-sulfonic acid, lilial (*p*-*tert*-butyl-*α-*methyl dihydrocinnamic aldehyde), and citralva (3,7-dimethyl-2,6-octadienenitile), as well as fatty acids such as myristic acid, palmitic acid, and stearic acid^42^. It has also been suggested that OBP2 is localized in the extracellular space^43^, which would be consistent with the present data indicating that OBP2 was secreted into the supernatant of cultured cells. Further investigation may help to clarify the species of odorants or lipids that bind to human OBP2B and the physiological processes that depend of their transport.

The present findings suggest that *ABO* and *OBP2B* are co-regulated in an epithelial cell-specific manner. Because OBP2B seems to be involved in physiological processes such as lactation, the regulatory element of *ABO* might play a pivotal role in the preservation of life or species. However, the physiological significance of ABO blood groups has not been clarified. Therefore, the biological role of the neighborhood gene controlled by the regulatory element of *ABO* might help to explain why the ABO gene has not been removed from the human genome during evolution.

## Methods

### Cells

We cultured the human gastric cancer cell line KATOIII (JCRB0611) and its derived clones B3 and B4 as described previously^6^. The human erythroleukemia cell line K562 (JCRB0019) and the human embryo fibroblast cell line OUMS-36T-1 (JCRB1006.1) were cultured as described previously^6^.

### RNA-seq

RNA-seq analyses were performed by DNA ChIP Research Inc as described previously^6^. The raw RNA-Seq data were deposited and released in GEO, with the GEO accession GSE169059.

### Quantitative PCR (qPCR)

RNA was prepared from the wild-type KATOIII cells and its derived clones B3 and B4, followed by cDNA preparation as reported previously^5,15^. Using real-time PCR with StepOne and SYBR Select (Thermo Fisher Scientific, Waltham, MA), the *ABO* and *β-actin* transcripts were quantified with gene-specific primers in accordance with the methods reported previously^21^, and the *OBP2B*, *SURF6*, *SURF1*, and *ADAMTS13* transcripts were quantified according to the manufacturer’s protocol for the RT^2^ qPCR Primer Assay (QIAGEN GmbH, Hilden, Germany). Quantification of those transcripts except for *β-actin* was also performed using QX200 Droplet Digital PCR (ddPCR, Bio-Rad Inc., Hercules, CA). The transcript of *18S rRNA* was also quantified by ddPCR with gene-specific primers according to the method reported previously^21^. Each 20 µL ddPCR reaction volume containing 10 µL of 2 × ddPCR EvaGreen SuperMix (Bio-Rad), 1 µL of cDNA diluted at 1:50, and 0.1 μM each gene-specific primer was prepared in a semi-skirted 96-well plate (Eppendorf AG, Hamburg, Germany). Following droplet generation on a QX200 droplet generator, the plate was sealed with PCR Plate Heat Seal, foil, pierceable (Bio-Rad), and PCR was carried out on a T100 or C1000 Touch thermal cycler (Bio-Rad). After PCR amplification, the plate was read using a QX200 droplet reader (Bio-Rad). QuantaSoft Analysis Pro software was used to assign positive/negative droplets and convert counts to copies/well. DNA fragments obtained by real-time PCR for *OBP2B* were then cloned into the pUC118 vector using a Mighty Cloning Reagent Set (Blunt End) (TaKaRa, Shiga, Japan). The nucleotide sequences of the amplified fragments were determined with a BigDye Terminator v1.1 Cycle Sequencing Kit (Thermo Fisher Scientific) with both M13 forward and reverse primers, and specific primers for the target. The sequencing run was performed on a SeqStudio Genetic Analyzer (Thermo Fisher Scientific).

### Reverse transcription (RT)-PCR

PCR amplification was carried out using primers OBP2B + 231 and OBP2B + 554 whose sequences were 5′-GAAAATCCTGATGCGGAAGA-3′ and 5′-GGTGGTAGGGTGGGCTCT-3′, respectively. The conditions for PCR were 94 °C for 3 min, 35 cycles of 98 °C for 10 s, 60 °C for 15 s, and 68 °C for 30 s, followed by incubation at 68 °C for 7 min. DNA fragments obtained from RT-PCR were then cloned into the cloning vector, followed by sequencing as described above.

### Western blot analysis

Whole-cell lysates were prepared from the wild-type KATOIII strain and its derived clones B3 and B4 using a Total Protein Extraction Kit (TaKaRa). Supernatant was prepared after the cells had been inoculated at 7 × 10^5^/ml into serum-free medium 72 h prior to harvesting, followed by concentration with Amicon^®^ Ultra-10K (Merck Millipore, Burlington, MA). Western blotting was carried out with rabbit anti-OBP2B monoclonal antibody (MA5-30722; Thermo Fisher Scientific) or anti-β-actin monoclonal antibody (017-24551; Wako, Osaka, Japan), followed by treatment with Amersham ECL Prime Western Blotting Detection Reagent (GE Healthcare, MA) and densitometry measurements with a LAS-3000 and MultiGauge v3.0 (FujiFilm, Tokyo, Japan).

### 5′-RACE

5′-RACE was performed using the SMARTer RACE 5′/3′ Kit (TaKaRa) in accordance with the manufacturer’s instructions. First-strand cDNA synthesis was primed using a modified oligo (dT) primer. After SMARTScribe Reverse Transcriptase (RT) reached the end of the mRNA template, it added several non-templated residues. The SMARTerIIA Oligonucleotide annealed to the tail of the cDNA served as an extended template for SMARTScribe RT. 5′-cDNA fragments were PCR-amplified using universal primer short and a gene-specific primer OBP2B + 237 whose sequence was 5′-GATTACGCCAAGCTTTCTGTGTCTGGTGGT-3′. The conditions for 5′-RACE cDNA amplification were 94 °C for 3 min, 40 cycles of 94 °C for 30 s, 68 °C for 30 s, and 72 °C for 3 min, followed by incubation at 72 °C for 10 min. The PCR products were electrophoresed through a 2% agarose gel, and DNA fragments were extracted using the MinElute Gel Extraction Kit (QIAGEN). The DNA fragments were then cloned into the cloning vector, followed by sequencing as described above.

### Plasmids

DNA fragments from − 1421 to − 41 or from − 552 to − 41 relative to the translation start site of *OBP2B* were obtained by PCR amplification using the genomic DNA prepared from KATOIII cells as a template, followed by subcloning at the *Kpn*I and *Nhe*I sites upstream of *luciferase* in the same orientation as that of *luciferase* in the pGL3 basic vector (Promega, Madison, WI) sequence in reporter plasmids OBP1.4 and OBP0.5, respectively. Deletion of the 5′-end of the upstream region of *OBP2B* was carried out by restriction enzyme digestion with *Apa*I, *Mbo*II or *Nco*I, followed by ligation into reporters OBP0.3, OBP0.2 or OBP0.1, respectively. The + 22.6-kb site was inserted into the *Bam*HI and *Sal*I sites downstream of *luciferase* to generate construct OBP0.3/C. The sequences of the inserts for all of the constructs used in this study were verified by detailed restriction enzyme mapping and DNA sequence analysis as described above. Plasmid DNA was purified using a HiSpeed Plasmid Maxi Kit (QIAGEN).

### Transfection and luciferase assay

Transient transfection of KATOIII cells was carried out using Lipofectamine LTX reagent (Thermo Fisher Scientific) with 1 μg of reporter plasmid and 0.001 μg of pRL-SV40 *Renilla* reporter in accordance with the manufacturer’s instructions. Transient transfection of K562 cells or OUMS-36T-1 cells was performed as reported previously^6^. After collecting the cells, cell lysis and luciferase assays were performed using the Dual Luciferase Reporter Assay System (Promega) to measure the activities of firefly and *Renilla* luciferases. Variations in transfection efficiency were normalized to the activities of *Renilla* luciferase expressed from the cotransfected pRL-SV40 *Renilla* luciferase reporter.

### Statistical analyses

All data are expressed as mean values and error bars representing standard deviation from at least three independent experiments. Data analyses for the two groups were performed using Student’s *t* test (*p < 0.05; **p < 0.01).

### Single-molecule fluorescence in situ hybridization

The wild-type KATOIII cells, and their derived mutant clones B3 and B4 were seeded on chamber slides at 50% confluency one day before the assay. RNA fluorescence in situ hybridization assays were performed with RNAscope technology utilizing the RNAscope Fluorescent Multiplex Kit V2 Kit (Cat # 323100, ACD, Hayward, CA, USA) in accordance with the manufacturer’s instructions. Briefly, the slides were rinsed once with phosphate-buffered saline (PBS), fixed with 10% neutral buffered formalin solution for 30 min at room temperature (RT), then dehydrated in an ethanol series and rehydrated in PBS. The slides were then treated with 15-fold diluted RNAscope protease III for 10 min at RT, followed by incubation with RNAscope probes for *ABO* and *OBP2B* (Cat #583991 and 584031-C2, respectively: ACD) for 2 h at 40 °C and stored overnight in 4 × SSC buffer. The probes were fluorescently labeled with Opal Dyes (Perkin Elmer, Waltham, MA; Opal570 diluted 1:500 and assigned to *ABO*; Opal520 diluted 1:500 and assigned to *OBP2B*) and stained with DAPI to label the nuclei.

### Image acquisition and signal quantification

The fluorescent images were acquired in the Z-series using a Zeiss LSM880 confocal microscope equipped with 40 × objectives. For each subject, five regions were selected randomly. Following image acquisition, in each region, the Z-series were compressed into one image using the “maximum intensity projection” method in ZEN black software, and the signal intensity was automatically adjusted by the Min/Max option, creating relative intensity. The images were then transferred to Fiji software for semi-quantitative analysis. First, based on the DAPI signals, the nuclei and background signals were separated based on an automatically selected threshold using the “Otsu” method in Fiji (version 1.53c)^44^. The overlapping nuclei were split by the “Watershed” function and each separated nucleus was assigned as a ROI. Then, the signals of *ABO* (Opal570) or *OBP2B* (Opal520) in each ROI were analyzed using the “Analyze Particle” function. Finally, the mean relative intensity of *ABO* (Opal570) or *OBP2B* (Opal520) in each ROI was measured and plotted. The regression curve and coefficient of determination (R^2^) were calculated in Excel using “data analysis” add-in.
